# AlO_6_ clusters’ electric storage effect in amorphous alumina supercapacitors

**DOI:** 10.1038/s41598-021-81483-2

**Published:** 2021-01-18

**Authors:** Mikio Fukuhara, Tomoyuki Kuroda, Fumihiko Hasegawa, Toshiyuki Hashida, Mitsuhiro Takeda, Kazuya Konno, Nobuhisa Fujima

**Affiliations:** 1 New Industry Creation Hatchery Center, Tohoku University, Sendai, 980-8579 Japan; 2 Fracture and Reliability Research Institute, Graduate School of Engineering, Tohoku University, Sendai, 980-8579 Japan; 3 National Institute of Technology, Sendai College, Natori, 981-1239 Japan; 4 Faculty of Engineering, Shizuoka University, Hamamatsu, 432-8561 Japan

**Keywords:** Energy science and technology, Materials science, Nanoscience and technology

## Abstract

In this study, the electric storage effect of AlO_6_ clusters in amorphous alumina (AAO) supercapacitors was investigated in terms of cluster morphologies under electron-beam irradiation. Based on first-principles density functional calculation, the optimised structure of AlO_6_ clusters around an O-vacancy is characterised by a large vacant space created by the absence of an O atom and its neighbouring Al atom. The localised electrons present near the two-atomic vacancies induce positive charges on the inside of the insulating oxide surface, ensuring the adsorption of many electrons on the surface. Electron-beam irradiation (adsorption) from 100 to 180 keV causes the lengths of the Al–O bonds of the cluster to shrink, but then return to the original length with decreasing voltage energy, indicating a rocking-chair-type charge-breathing effect accompanied by a volume expansion of approximately 4%. The *I–V* and *I–R* characteristics depicted Coulomb blockade for the switching effect of both the negative and positive potentials. The Ragone plot of the AAO supercapacitor is located at capability area of the second cell.

## Introduction

The most popular electrochemical capacitor is an electric double-layer supercapacitor (SC) where the interfaces of high specific-area materials such as porous carbon materials or porous metal oxides of some metals are charged and discharged by ion or radical diffusion^1–3^. In sharp contrast to the conventional SC, we developed physical dry ones based on both the quantum-size effect and the offset effect of the positive charges on uneven insulating material surfaces. An amorphous aluminium-oxide (AAO) device can store a large amount of electric charge as a supercapacitor with various electrical applications. The storage potential of AAO is given by the presence of AlO_6_ clusters with O-vacancy sites on its blackish, nanometre-sized, uneven Al_2_O_3−x_ surface^4,5^. Although the six-coordinated AlO_6_ species are minor components, we could fabricate the AAO film of an AlO_6_ octahedron on the aluminium surface through the anodic polarisation process with nanometre-sized bubbles of carbon oxide^6^, using AlY_10_ amorphous alloy ribbons^7^. The film indicated a switching effect for both positive and negative potentials in air, demonstrating the potential of using rechargeable dry solid supercapacitors instead of practical Li ions. To explain why the supercapacitor featured superior electric storage, we first demonstrated the extremely enhanced electron trapping due to both the quantum-size effect and the offset effect caused by positive charges at the oxygen-vacancy sites.

Our current interest lies in studying the electrostatic role of induced electrons in the structural morphology of AlO_6_ clusters for superior electric storage. In this study, we first analyse cluster morphology as a function of applied electron-beam irradiation with the help of the first-principles simulation (molecular dynamics calculation) method. We assume that the cluster morphology with atomic vacancies under electron-beam radiation (adsorption) provides useful information for the interpretation of the electron charging mechanism of AlO_6_ clusters. Nakamura et al.^8^ reported that the crystallisation of AAO can be attributed to electron-beam irradiation. However, to the best of our knowledge, no prior research has been conducted on the electron-induced structural morphology of AlO_6_ clusters in the context of supercapacitors. Cluster morphology, with atomic vacancies, provides new insights into the electronics of nanometre-sized clusters.

## Results and discussion

### Structure change of AlO_6_ clusters under electron-beam irradiation

**Figure 1:**
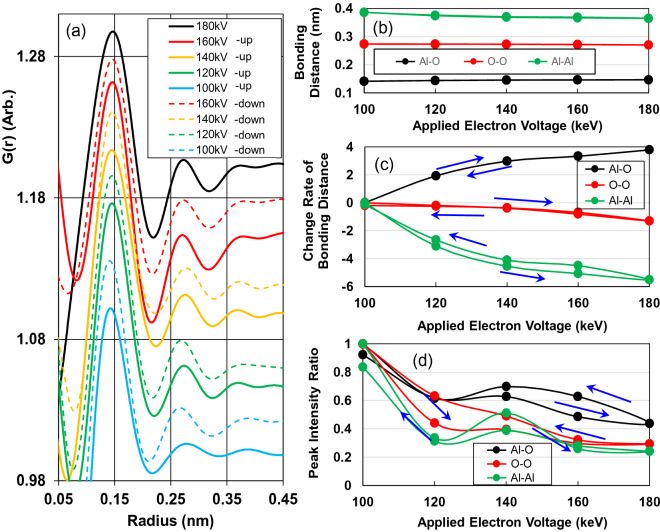
(**a**) Changes in atomic pair distribution functions (PDFs) under irradiation at 100, 120, 140, 160, and 180 keV with the dose rate of 6 nA/m^2^ with increasing and decreasing voltage energy runs. (**b**) Bonding distances, (**c**) change rates of distance, and (**d**) intensity ratios for peaks of Al–O, O–O, and Al–Al bonds as functions of applied voltage.

Because electron radiation at high dose rates of 10^17^ e/m^2^s crystallises AlO_6_ clusters (see Supplementary Information S2), we investigated the cluster morphology of AlO_6_ clusters at 100–180 keV, with a dose rate of 10^9^ e/m^2^s, in terms of the electron-charging mechanism. Figure 1a shows the changes in atomic pair distribution functions (PDFs) under irradiation at 100–180 keV in increasing and decreasing voltage energy runs. The intensity–peaks of the Al–O, O–O, and Al–Al bonds can be observed at approximately 0.15, 0.27, and 0.38 nm, respectively. The lengths of the Al–O and O–O bonds are smaller, and those of the Al–Al bonds are larger than the lengths obtained experimentally or through simulated PDFs of amorphous alumina composed of AlO_4_ or AlO_5_ clusters^9–12^. The bonding distances and change rates of distance for the Al–O, O–O and Al–Al bonds as functions of applied voltage energy are presented in Fig. 1b and c, respectively. Compared with the shrinkage of the O–O and Al–Al bonds, the length of the Al–O bond increases by as much as 4% and then returns to zero as the applied voltage energy increases from 100 to 180 keV and decreases to 100 keV. Because the first peak of Al–O is affected by insufficient electron intensity, we considered only the O–O and Al–Al peaks in this study. The peak intensity ratios of 120, 140, 160, and 180 keV to peak intensity of 100 keV (Fig. 1d) approximately decrease with increasing applied voltage energy up to 180 keV and then increase when the applied voltage energy is decreased to 100 keV. However, none of the peaks overcome the peak intensity at 100 keV. Because crystallisation of γ–Al_2_O_3_ with a long-period structure is accompanied by an increase in the peak intensity of the O–O and Al–Al bonds^9^, the structural morphology of the AlO_6_ cluster is stable against electronic excitation up to 180 keV. Yong et al.^13^ also reported that amorphous alumina separated O^2−^ gas bubbles when it was irradiated at an energy of 200 keV and a beam current of 5.4 × 10^–10^ A. Thus, the critical voltage energy of 180 keV is higher than those of 100 and 150 keV for metal (Na, Li, K, Pb, and Ag) β-alumina^14^ and silicon nitride^15^, respectively.

### Optimised structure of AlO_6_ clusters and its electronic role

**Figure 2:**
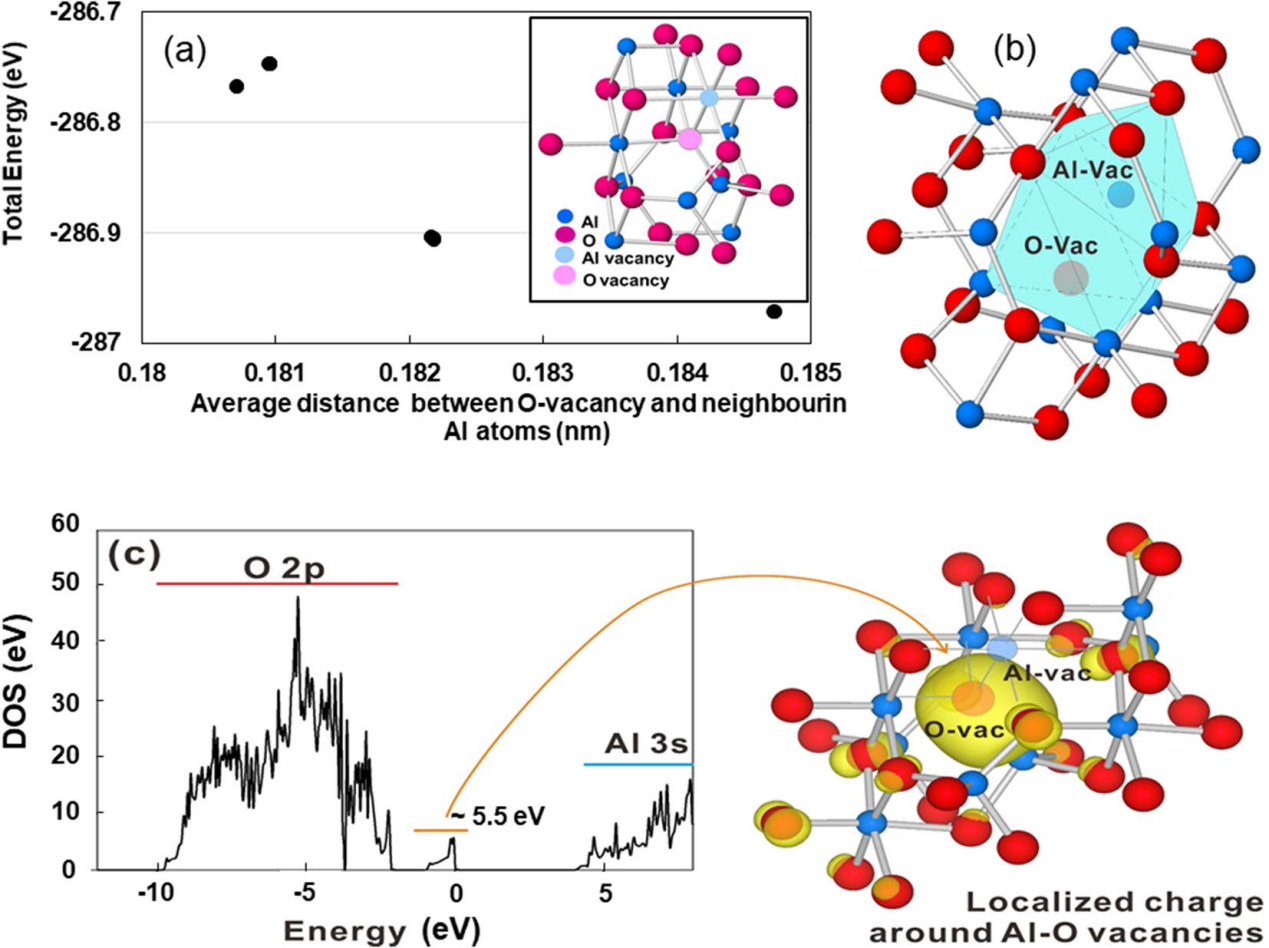
(**a**) Relation between energy and atomic distance between O vacancy and its neighbouring Al vacancies. Inset in (**a**): Optimised structure of AlO_6_ cluster with oxygen and aluminium vacancies. (**b**) Vacant cage produced by deficit of an O atom and its neighbouring Al atom in AlO_6_ cluster. (**c**) Density of state for O 2*p* and Al 3*s* in AlO_6_ cluster. Isolated electronic state locally occurs in vicinity of two-atom vacancies.

Next, we simulated the optimised structure around the AlO_6_ clusters. We derived the relationship between energy and average atomic distance between the O vacancy and the vacancies in the neighbouring Al atoms using the molecular dynamic method. The calculated results are presented in Fig. 2a. The larger the average distance, the higher the energy required for stability, thereby bringing the neighbouring O atoms closer. Thus, we can optimise the local structure of the AlO_6_ cluster unit (Al_2_O_2.875_ with a density of 2.79), which has a shortage of neighbouring O and Al atoms, as depicted in the inset in Fig. 2a. We then simulated the density of states (DOS) for the O 2*p* and Al 3*s* electrons of the AlO_6_ cluster. Figure 2c depicts an isolated electronic state in the band gap, which locally occurs in the vicinity of the O vacancy and its neighbouring Al vacancies. The existence of an electronic state in insulating oxide clusters provides new insights into the electronics of amorphous oxides with nanometre-sized clusters. Indeed, amorphous titanium-oxide (ATO) supercapacitors are characterised by electrostatic induction of a large positive charge on the uneven oxide surface based on the electronic conduction state derived from TiO_2_/VO_2_ nanostructural interfaces in the oxide^16^. The cage structure consists of a vacant space of 0.0214 nm^3^, as represented in Fig. 2b.

### An electric storage system of AlO_6_ clusters

**Figure 3:**
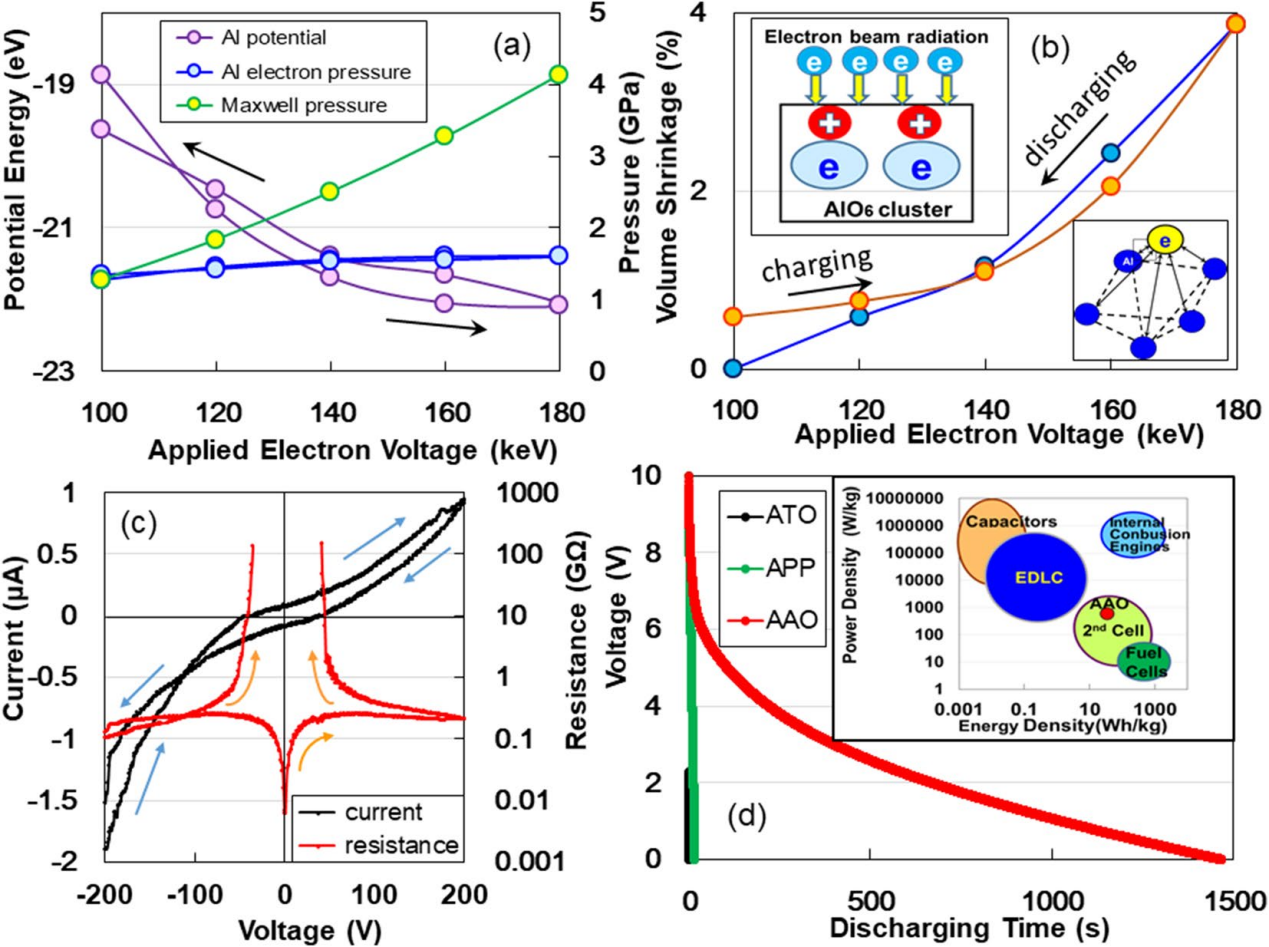
(**a**) Al potential, Al electron pressure and Maxwell stress, and (**b**) volume shrinkage as a function of applied electron voltage energy. Upper inset in (**b**): Schematic shrinkage model explaining huge electric storage for AlO_6_ cluster with two atom vacancies. Lower inset of (**b**): Shrinking model of AlO_6_ cluster by electrostatic attraction force among Al atoms induced by screened electrons. (**c**) *I–V* and *R–V* characteristics from − 200 to + 200 V. (**d**) Charging/discharging behaviours for ATO, APP, and AAO. Inset in (**d**): Comparison of power density and energy density for AAO (this study), APP, EDLC, batteries, fuel cells, and internal combustion energies (based on Whittingham’s study^22^).

From the shrinkage of the Al–Al bond length under irradiation energies of 100–180 keV, as depicted in Fig. 1c, we calculated the electrostatic potential and electron pressure of Al and the electrostatic compressive pressure induced by electron adsorption as a function of applied irradiation energy (see Supplementary Information S6). The results are presented in Fig. 3a. The electron pressure of the Al atom gradually increases and decreases as the applied electron energy increases up to 180 keV and returns to 100 keV, respectively, while the electrostatic potential decreases to – 22 eV at 180 keV and returns to − 19.7 eV at 100 keV. Here, we considered the Maxwell stress (electric field stress), which performs mechanical work in contracting the thickness of the vacancy cages (Fig. 2b) enclosing the two atomic vacancies in the AlO_6_ cluster. Maxwell compression increases from 1.3 to 4.1 GPa, accompanied by a volume shrinkage of nearly 4% (Fig. 3b), according to applied electron voltage energies (see Supplementary Information S4), and returns to 1.3 GPa at 100 keV. From the measurement of bulk modulus for the AlO_6_ cluster, we indeed obtained 4.1 GPa using ∆V/V at 180 keV, where ∆V is the change in volume V (see Supplementary Information S5). Here, it should be noted that lattice contractions have been observed for fine metallic particles such as silver^17^, copper, platinum^18^, and gold^19^. This is indicated by the increase in electrostatic force induced by electron screening^20^. Hence, we infer by analogy that electron-beam irradiation (adsorption of electrons) causes the AlO_6_ cluster to shrink owing to the electrostatic attraction force induced by the screened-electrons among the Al atoms (lower inset in Fig. 3b). The schematic for electro adsorption induced by electron-beam irradiation is presented in the upper inset of Fig. 3b. The localised electrons present near the two-atomic vacancies in the AlO_6_ cluster induce positive charges on the inside of the insulating oxide surface, resulting in the adsorption of many electrons under electron-beam irradiation. However, when the value of the applied irradiation energy returned to 100 keV, we obtained the same PDF as that observed at the starting voltage of 100 keV. This indicates the desorption of electrons due to the volume recovery of the vacancy cage. The AlO_6_ cluster seems to have a ‘rocking -chair-type’ electric storage system similar to a breathing lung. The amorphous materials of interest can be distinguish between ‘dry’ body and ‘wet’ cells such as electric double-layer capacitors (EDLCs) and secondary cells, which are controlled by ion diffusivity.

### Electricity switching effect of rechargeable dry solid supercapacitors

Our study presents further evidence of large electron storage values for rechargeable dry solid supercapacitors using the AAO device. Figure 3c represents the double *I*–*V* and *R*–*V* characteristics obtained by DC current method between – 200 and + 200 V in air at 293 K. The *I*–*V* curves indicate nonlinear electronic transport behaviour, which is similar to the Coulomb blockade behaviour observed in a metal–semiconductor junction which is characterized by a Schottky junction^21^. We can see the ‘Coulomb gap’ depicted by the hyperbolic curves in the *R–V* characteristics. The *I*–*V* curves are asymmetric with respect to zero bias. Although the asymmetry could be generally attributed to the specimen size^22^, the current *I* reached zero at – 35.6 and + 35.6 V upon increasing and decreasing the applied voltage *V*, respectively. Zero current at – 35.6 V corresponds to the emission of electrons from the negative electrode to the convex portions, whereas zero current at + 35.6 V indicates the emission of electrons from the concave portions to the positive electrode, as described in our previous paper^5^. This is further evidence of the electricity switching effect of rechargeable dry solid supercapacitors.

Figure 3d depicts the discharging behaviour of AAO, ATO, and amorphous perfluorinated polymer (APP) under a constant current of 1 nA after charging by DC currents of 1 mA for 240 s. The discharging curves do not match the standard trace, which is typical for conventional supercapacitors. This can be explained by electroadsorption^23^ occurring on nanometre-sized capacitors. The discharging time of AAO is approximately 3000 and 100 times longer than those of ATO^7^ and APP^24^, respectively.

Since the discharging curve for AAO in Fig. 3d indicates an electric power of 2.43 × 10^–9^ W and electric energy of 9.90 × 10^–10^ Wh, we obtained a power density of 115.4 W/kg and an energy density of 47.0 Wh/kg (see Supplementary Information S7). The Ragone plot, the relation between energy density and power density, for the AAO supercapacitor is presented in the inset of Fig. 3d, along with conventional capacitors, EDLC, and the second fuel cells^25^. The plot of the AAO supercapacitor is located at capability area of the second cell. Thus, we can apply rechargeable dry supercapacitors instead of practical Li ions in a liquid solvent. The quick, powerful, and energy-rich storage effects for the AAO of interest are promising for future electronic devices and electric power applications such as hybrid electric vehicles and backup power supplies^1^.

### Complex evaluation of electric storage

**Figure 4:**
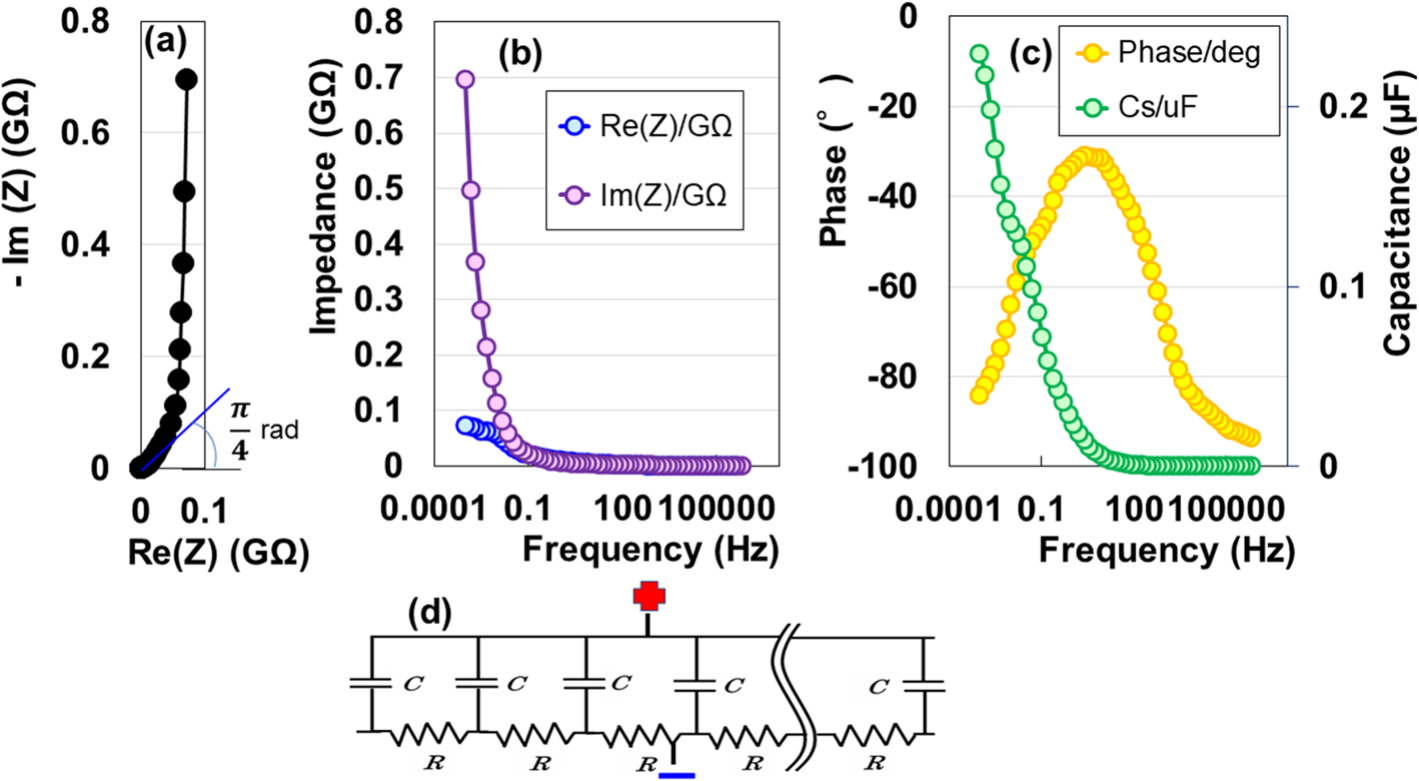
(**a**) Nyquist plot, (**b**) Bode plot, and (**c**) phase and series capacitance as a function of frequency for AAO device. (**d**) Electric distributed constant equipment circuit organized on the AAO surface.

To analyse non-destructively the electrostatic contribution of the specimen, we finally performed the AC impedance measurement from 1 mHz to 1 MHz using Nyquist and Bode diagrams at 293 K. We present a complex-plane (Nyquist) plot of the impedance data in Fig. 4a. The frequency dependent impedance is characterized by the combined pattern of a line with a slope of π/4 rad and a straight vertical line. The π/4 rad region (Warburg region) indicates the distributed resistance/capacitance in the porous electrode^26,27^. This suggests that the electrode is an AAO film with a porous surface with a high resistance. A nearly vertical line for the impedance data is produced by a series RC circuit, as well as a graphene EDLC^28^. The rapid increases in imaginary impedance compared with the real impedance, in the lower frequency region of the Bode diagram (Fig. 4b), suggests an evidence for DC charging. Moreover, the decrease in the phase angle to − 90° with decreasing frequency indicates another evidence of DC charging (Fig. 4c). Thus, the AAO device offers a nearly ideal electric distributed-constant structure for electric storage (Fig. 4d). The series capacitance was 0.23 μF at 1 mHz (Fig. 4c).

## Methods

The fabrication of AAO specimens and analyses of the charging/discharging behaviour were reported in previous papers^4–6^. Electron-irradiation was performed using JEOL (JEM-2100) transmission electron microscopy (TEM) at 100–200 keV. Electron diffraction patterns, on a fluorescent screen, created from an irradiated area under an electron density of 10^9^ e/m^2^s were evaluated by radial distribution function analysis^29^. The optimised local atomic configurations of the AlO_6_ clusters were determined through a plane-wave-based first-principles density functional calculation (VASP 5.3)^30^ in a γ-alumina-based system with O vacancies.

## Supplementary Information

Supplementary Information.
