# Supplementary material for: AlO6 clusters’ electric storage effect in amorphous alumina supercapacitors
Source: Sci Rep. 2021 Jan 18;11:1699. doi: 10.1038/s41598-021-81483-2 (PMC7813864; doi:10.1038/s41598-021-81483-2)
Supplement: Supplementary file 1 — Supplementary Information. [file 41598_2021_81483_MOESM1_ESM.docx]

**SUPPLEMENTARY INFORMATION**

**AlO_6_ clusters’ electric storage effect in amorphous alumina supercapacitors**

Mikio Fukuhara^1^, Tomoyuki Kuroda^1^, Fumihiko Hasegawa^1^, Toshiyuki Hashida^2^, Mitsuhiro Takeda^3^, Kazuya Konno^3^ and Nobuhisa Fujima^4^

^1^ New Industry Creation Hatchery Center, Tohoku University, Sendai, Japan, 980-8579

^2^ Fracture and Reliability Research Institute, Graduate School of Engineering, Tohoku University, Sendai, Japan, 980-8579

^3^ National Institute of Technology, Sendai College, Natori, Japan, 981-1239

^4^ Faculty of Engineering, Shizuoka University, Hamamatsu, Japan, 432-8561

**S1. Methods**

The blackish AAO specimens were prepared by using the anodic polarisation process in a 5 wt. %H_2_SO_4_ solution at 16 V and 278 K, with AlY_10_ alloy ribbons prepared by melt spinning technique, using a single-wheel melt-quenching apparatus. The charging/discharging behaviour of the specimen was analysed using galvanostatic charge/discharge through a potentiostat /galvanostat with dc voltage of 10 V and current 1 mA for 1,500s at room temperature, with a complex impedance between 1 mHz and 1 MHz and 10 mV. Current-voltage (*I-V*) and resistivity-voltage (*R-V)* characteristics were measured by DC voltages from -200 to 200 V in air at a sweep rate of 1.24 V/s, using a Precision Source Measure Unit (B2911A, Agilent).

**S2. Void formation and crystallisation by long-term and strong electron-irradiation on AlO_6_ cluster surface**

Fig. S1 The ADF-STEM images before (a) and after (b) EDS analysis with beam current density of 1.6 nA/m^2^ for about 60 sec. at 160 kV.

When the concentrations of Al, Y and O on the specimen were determined using energy disperse X–ray spectroscopy (EDS) attached with JEOL JEM2100 with beam current density of ~1.6 A/m^2^ for around 60s at 160 kV, we observed voids of 80/nm^2^ in ADF-STEM images after EDS analysis. The ADF-STEM images before and after EDS analysis are shown in Fig. S1 (a) and (b), respectively. This means that the long-term electron radiation destroys AlO_6_ clusters, *i.e*., actually filliping of atoms. For stability of AlO_6_ claster for electron iradiation, we observed crystallization under electron irradiation at the dose rate of 10^17^ e/m^2^s as shown in Fig. S2. The strong electro-beam irradiation accelerated crystallisation of AlO_6_ clusters.

Fig. S2 TEM images ((a) and (c)) and SAED analyses ((b) and (d)) under the irradiation energy of 180 keV with the dose rate of 10^9^ and 10^17^ e/m^2^s, respectively.

S3. **Volume calculation of a cage (Fig. 2b)**

The volume of the cage (0.0214 nm^3^) in Fig.2b is obtained by dividing the optimized structure of the cage into some tetrahedra and summing up volumes of the tetrahedra. To estimate the change of the cage volume, we employ the following simplified model of the cage (Fig. S3):

If a (b) is taken to be the length of Al-O (Al-Al or O-O) bond, the volume V_1_, V_2_ and V_3_ surrounded by frames of black, pink and green lines, respectively are given by

V_1_ = $\frac{1}{3}$ab^2^ (S1)

V_2_ = $\frac{1}{2}$ab^2^ (S2)

V_3_ = $\frac{1}{3}$ab$\sqrt{a^{2}+b^{2}}$ (S3)

Thus, we find the cage volume V.

V = V_1_ + V_2_ + V_3_ = $\frac{1}{3}$ab$\left( \frac{5}{2}b+\sqrt{a^{2}+b^{2}} \right)$ (S4)


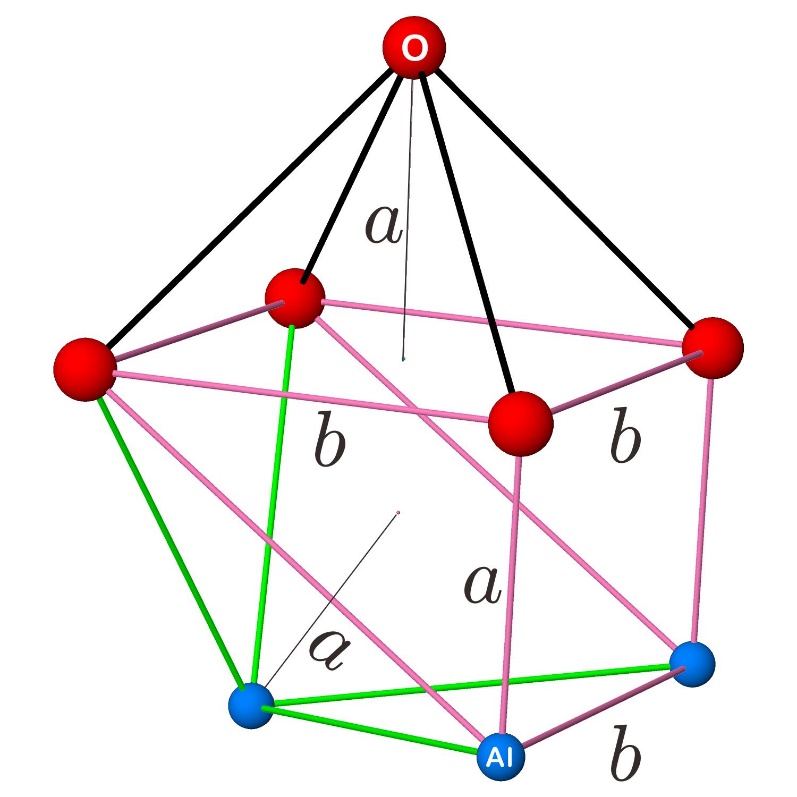
The cage volumes for applied electron irradiation voltages are presented at Table S1.

Fig. S3 A cage divided by three parts V_1_, V_2_ and V_3_ surrounded by frames of black, pink and green lines, respectively.

**S4. Compressive force acting on an interface of a cage (Fig. 2b) when strong electron beams irradiate on the surface of AlO_6_ cluster**

When the localised electrons occurred near the three-atomic vacancies in AlO_6_ cluster induce positive charges at inside of insulating oxide surface, a compressive electric field stress (Maxwell stress) is given by the following equation^27^,

P = $\frac{1}{2}ɛE^{2}$, (S5)

*ɛ* = $ɛ_{0}$*ɛ_Al_* (S6)

where *E*, ɛ and $ɛ_{0}$ are electric field strength for thickness of 15 μm (Ref. 8), permittivity and permittivity of free space (8.85 4×10^12^ F/m), respectively. $ɛ_{Alumina}$ is permittivity of alumina (6.5, Ref. 28). From Eqs. (S5), (S6) and Table S1, we can calculate the compressive stress when applied electron irradiation voltage is 180 keV:

P = $\frac{1}{2}ɛE^{2}$ = $\frac{1}{2}{ɛ_{0}ɛ_{Al}E}^{2}$ = $\frac{6.5\times8.854 \times{10}^{-12}\times{{(1.2\times10}^{10})}^{2}}{2}$ = 4.144 GPa. (S7)

Table S1 presents compressive stresses at voltage region from 100 to 180 keV, along with corresponding *E*.

Table S1 Al-O bond length, electric strength, Maxwell stress and cage volume for applied electron irradiation voltage energy from 100 to 180 keV

| Irradiation energy (keV) | 100 | 120 | 140 | 160 | 180 |
| --- | --- | --- | --- | --- | --- |
| O-O length (nm) | 0.2788 | 0.2733 | 0.2728 | 0.2716 | 0.2702 |
| Electric strength (V/m) | 6.66×10^9^ | 8.0×10^9^ | 9.33×10^9^ | 1.067×10^10^ | 1.2×10^10^ |
| Maxwell stress (GPa) | 1.279 | 1.842 | 2.506 | 3.274 | 4.144 |
| Cage volume (nm^3^) | 0.0169 | 0.0168 | 0.0167 | 0.0165 | 0.0162 |

**S5.　Measurement of bulk modulus and calculation of compressive pressure under applied electron irradiation for AlO_6_ cluster**

Seven kinds of elastic parameters (Young *E*, shear *G* and bulk *B* modulus, Lamé parameter λ, Compressibility β, Poisson ratio ν, anisotropy factor *A(=*$\sqrt{3}$*Vs/V_l_*) and longitudinal *V_l_* and transverse *Vs*) velocities of AlO_6_ cluster were measured by un ultrasonic measuring system (TP-1001, Toshiba Tungaloy) at room temperature, using longitudinal and transverse waves with a frequency of 5 MHz.^29, 30^ These data are presented at Table 2.

Because the volume strain is equal to the change in volume, Δ*V*, divided by the original volume, *V*, we can characterize a volume compression in terms of the bulk modulus, B,^31^ defined as

B = —$\Delta P/ \frac{\Delta V}{V}$ . (S8)

Taking $\frac{\Delta V}{V}=-0.0397$ and *B* =103.3 GPa, we get positive pressure of 4.1 GPa.

Table 2

| longitudinal velocity *V_l_* | 8375 m/s |
| --- | --- |
| transverse velocity *Vs* | 4005 m/s |
| Young modulus *E* | 91.9 GPa |
| Shear modulus *G* | 34.0 GPa |
| Bulk modulus *B* | 103.3 GPa |
| Lamé parameter λ | 80.7 GPa |
| Compressibility β | 0.00977 1/GPa |
| Poisson ratio ν | 0.352 |
| anisotropy factor *A* | 0.828 |

**S6. Calculating　the electrostatic potential of Al on a nanometre-sized uneven surface using the Thomas-Fermi statistic method**

Our interest lies in studying the electrostatic adsorption of Al and O atoms surrounding the AAO with the nanometre-sized uneven surface with the quantum-size effect in view of the Thomas-Fermi (TF) electronic screening theory. The TF model has been applied to approximate calculations of potential fields and charge densities in elements as a function of lattice spacing. To the best of our knowledge, however, no detailed investigation has been conducted into the quantum-size effect for electric storage.

In general, nanoparticles with a particle size below 100 nm are characterised by a significant paucity in the ratio of chemical bonds in the particle surface. This suggests an increase in free electrons coming from the outer *s* and *p* subshells and resulting in a relative decrease in the inner subshells in nanoparticles. This physical picture explains the lattice expansion of nanoscale compound particles from the neutralization of the screening effect cause by the decreased binding-electron ratio^32^. By reverse analogy, then, we can calculate the electrostatic potential and the electronic pressure of Al and O atoms surrounding the AAO.

When we assume that the convex portion is almost a half-sphere, the ratio *η* of the topmost atomic layer volume *V*’ to the half-sphere volume *V* increases as the half-sphere diminishes in size:

*Η* = *V’/V*= 2*πR^2^r/*(4/6)*πR^3^ =* 3*r/R,* (S9)

where *r* and *R* are radii of the layer atom and the sphere, respectively. Because the outermost bonding of the surface atoms is missing, the missing electrons are apparently free. The ratio of free electrons to binding electrons increases as the size decreases, suggesting a relative decrease in the binding electrons. Because the free electrons squeeze into the inner binding-electron region in nanometre-sized metallic particles^33^, the density *ρ* of the electrons associated with rigid bonding is calculated as follows:

*ρ* ＝ *ρ_0_* (1+*η*), (S10)

where *ρ_0_* is the electron density of the “bulk” atom.

The following relation derives the screening length 1/*λ*^34^:

*λ^2^* = 4*ρ*^1/3^*/a_o_,* (S11)

where *a_o_= h*^2^*/ me*^2^ is the radius of the first Bohr orbit of a hydrogen atom. According to TF approximation, the screened Coulomb potential *φ*(r) is written in the following form:

*φ*(r) = *qe^-λr^*/*r*. (S12)

When the screened Coulomb potential, the screening length, the electron density, and the atomic radius of the “bulk” and nanosphere atoms is *φ_o_*, 1/*λ_o_, ρ_o_, r_o_*, and *φ_1_*, 1/*λ_1_, ρ_1_, r_1_*,

respectively, we can solve

*φ*(*r_o_*) = *φ*(*r_1_*) (S13)

for *r_1_* under an electrostatic equilibrium in potential at *r_o_* and *r_1_*, using the T-F table^32^*φ_0_*(*x*).

We then consider an electronic contribution for the electric storage in terms of the bonding character of Ai and O ions, using their electrostatic potential. Because outer electrons in Al and O ions are unsettled in the electronic structure with discrete permitted energies, several free electrons can move around the Al and O ions. According to ideal gas model, the pressure of the material arises almost entirely from the electrons, both because they outnumber the nuclei and because of their relatively small mass^35^. In electronic kinetic theory, the relation between electronic pressure *P* and the total number of electrons per unit volume *n* is given as follows:

*P* = $\frac{1}{5}({\frac{3}{8\pi})}^{\frac{2}{3}}\frac{h^{2}}{m}n^{\frac{5}{3}}$. (S14)

The number *n* is related to effective potential energy *U* of the outer electrons as follows:

*n* = $\frac{8\pi}{3h^{3}}{(-2mU)}^{\frac{3}{2}}$. (S15)

Eqs. (S13) and (S14)can then be combined as follows:

*P* = $\frac{8\pi}{15h^{3}}m^{\frac{3}{2}}{(-2U)}^{\frac{5}{2}}$, (S16)

where *h* is a Plank constant and *m* is the mass of the electron.

From Eqs. (S12) and (S15), we can calculate the electrostatic potential and the induced outer electronic pressure of the Al and O atoms surrounding the AAO. We used 0.0535 (Ref. 36) and 0.140 nm (Ref. 37) as the ionic radius of Al and O.

**References**

27. M.H. Sadd, Displacement Potentials and Stress Functions in Elasticity (Elsevier, New York, 2005), p. 347-369.

28. Khosa, R. Y., Thorsteinsson, E. B., Whinters, M., Rorsman, N., Karhu, R., Hassan, J. and Sveinbjörnsson, AIP Advances. **8**, 025304 (2018).

29. Fukuhara, M., Yagi, M. and Matsuo, A., Temperature dependence of elastic parameters and internal frictions for the TiNi alloy, *Phys. Rev*., B65, 224210 (2002).

30. Fukuhara, M., Inoue A. and Nishiyama, N., Rubberlike entropy elasticity of a glassy alloy, *Appl. Phys. Lett*., **89**, 101903 (2006).

31.Serway, R. A. Physics for Scientists & Engineers with Modern Physics, 3rd edition, (Saunders Colledge Publishing, Chicago, 1990), p.311.

32. Kittel, C. *Introduction to Solid State Physics*, 4th edition (Wiley, 1971), p.279.

33. Fukuhara, M. Lattice expansion of nanoscale compound particles. *Phys. Lett. A.* **313**, 427-430 (2003).

34. Condon, E. U. & Odabaşi, H. *Atomic Structure* (Cambridge University Press, London, 1980), p.454.

35. Hamann, S. H. *Physico-Chemical Effects of Pressure* (Butter Worths Scientific, London, 1957), p. 59.

36. Kittel, C. *Introduction to Solid State Physics*, 4th edition (Wiley, 1971), p.76.

37. Shannon, R. D. Revised effective ionic radii and systematic studies of interatomic distances in halides and chalcogenides. *Acta Cryst*. **A32**, 751-767 (1976).
